# Supplementary material for: Are carbon emissions trading and green financial instruments synergistic? -Comprehensive quantitative research based on content analysis
Source: PLoS One. 2024 Mar 7;19(3):e0298601. doi: 10.1371/journal.pone.0298601 (PMC10919601; doi:10.1371/journal.pone.0298601)
Supplement: S1 Data — (ZIP) [file pone.0298601.s001.zip › Data/地级市Z_F工作报告.zip!/║╙─╧/╓ú╓▌╩╨/└┤╫╘╣1⁄2╓┌║┼í╛┬φ┐╦╩2╛▌═°í┐_2009.docx]

各位代表：

现在，我代表市人民政府，向大会作政府工作报告，请予审议，并请各位政协委员提出意见。

过去五年工作回顾

本届政府任期的五年，是我市在解放思想中谋求科学发展，在克难攻坚中推进跨越式发展，在统筹兼顾中促进和谐发展的五年。面对纷繁复杂的经济形势和竞争激烈的发展环境，市人民政府在省委、省政府和市委的正确领导下，坚持以邓小平理论和“三个代表”重要思想为指导，深入贯彻落实科学发展观，紧紧依靠全市人民，抢抓机遇求突破，凝心聚力抓落实，全面完成了市十二届人大确定的各项目标任务，荣获国家卫生城市、国家园林城市、全国绿化模范城市、全国科技进步先进城市、全国双拥模范城市和全国创建文明城市工作先进城市等称号，踏上了建设国家区域性中心城市的新征程。

——综合实力迈上新台阶。全市生产总值由2003年的1074亿元增加到3004亿元，年均增长15.2%。地方财政一般预算收入由65.9亿元增加到260.4亿元，年均增长31.6%；地方财政一般预算支出由2003年的84.2亿元增加到288.8亿元，年均增长28%。县域经济和城区经济实力明显增强，有5个县（市）经济总量位列全省十强，有6个县（市）区地方财政一般预算收入突破10亿元。

——农业、工业和服务业持续发展。粮食生产连续5年保持增长，2008年单产创历史最高水平，总产量达到165.2万吨。规模以上工业增加值达到1223.7亿元，是2003年的2.8倍，年均增长22.6%。服务业增加值达到1249.8亿元，是2003年的1.8倍，年均增长12.9%。二、三产业占生产总值的比重达到96.8%。

——投资、消费和进出口快速增长。全社会固定资产投资达到1772.7亿元，社会消费品零售总额达到1206.3亿元，进出口总额达到42.8亿美元，分别是2003年的3.5倍、2.3倍和3倍。

——城乡面貌发生显著变化。建成区面积由2003年的212.4平方公里扩大到302平方公里，城镇化率由57%提高到62.3%，建成区绿化覆盖率、绿地率、人均公共绿地分别达到33.9%、32%和9.1平方米，城区环境空气质量二级以上天数由308天提高到325天。农村生活条件和居住环境明显改善，公路总里程由6593公里增加到10898公里，电话、有线电视和自来水普及率大幅提升。

——社会事业全面进步。公共财政用于教育、科技、文化、卫生等社会事业的支出年均增长31.4%。科技创新能力不断提高，进入国家知识产权示范创建市行列，并成功跻身中国城市综合创新力50强。实现了城乡免费义务教育。在全国率先推行了市属国有破产和困难企业退休人员大病统筹医疗保险。在全国省会城市中率先建立了城乡居民养老保险制度。城镇居民基本医疗保险工作被国务院列为全国首批试点城市。惠及全民的教育、医疗等民生保障体系初步形成。

——城乡居民生活水平明显提高。城镇居民人均可支配收入和农民人均纯收入分别达到15732元和7548元，分别是2003年的1.9倍和2.1倍。年末城乡居民储蓄存款余额达到2067.2亿元，是2003年的2倍。每万人拥有医疗床位52张、拥有卫生技术人员57人。城镇居民和农村居民人均住房面积分别由22.4平方米、39.7平方米增加到26平方米和46.4平方米。城镇居民和农村居民恩格尔系数分别为34.8%和31.3%。

这些成就，标志着我市经济社会发展发生了重大变化，标志着我市全面建设小康社会迈出了坚实步伐。五年来，我们所做的主要工作是：

一、牢固树立科学发展观，全面实施跨越式发展战略

以科学发展观统揽经济社会发展全局，认真贯彻落实中央领导视察河南、郑州时的指示精神，按照省委、省政府提出的中原崛起看郑州、提升郑州“六个力”、在全省发挥带头作用的要求，进一步解放思想、拉高坐标，我市于2006年制定并全面实施了经济社会跨越式发展战略，成立了八个重点工程指挥部，建立了一个项目、一套班子、一抓到底的“三个一”工作机制和一系列工作制度，以扶优扶强、产业园区建设、科技自主创新、新农村建设、城市建设管理、交通枢纽和物流、文化建设、生态建设等八项重点工程为依托，以280个重点项目和22个重大发展项目为抓手，实施了一大批带动能力强、发展后劲足的重大产业项目，“三个一千、三个翻番”的三年跨越式发展目标和“五个二”、 “31245”、“五个四”等年度奋斗目标圆满实现。共实施省市重点项目643个，累计完成投资1977亿元，年均增长44.1%。跨越式发展战略的实施，提升了发展实力，激发了发展潜力，成为我市贯彻落实科学发展观的成功实践。

二、优化工业结构，打造先进制造业基地

坚持走新型工业化道路，推动工业结构优化升级，汽车、装备制造、铝、食品和能源工业等优势行业的竞争力进一步增强。全部工业完成增加值1484.7亿元，是2003年的2.4倍，年均增长19.1%，占全市生产总值的比重由40.7%提高到49.4%。规模以上工业企业销售收入和利润分别达到4260亿元和443亿元，分别是2003年的4.9倍和8.4倍。年销售收入超亿元企业增加853家，达到998家，超百亿元企业实现“零”的突破，达到7家。

年销售收入超50亿元园区达到16个，其中超百亿元园区达到9个。五年累计完成工业投资1877.5亿元，新增汽车产能15.5万辆、食品加工能力167.8万吨、铝加工能力126万吨、氧化铝产能70万吨，中铝河南分公司氧化铝产量位居亚洲第一，宇通公司城市豪华客车和城市公交车国内市场占有率位居第一，速冻食品年产量稳居全国第一。全市高新技术产业实现增加值410亿元，是2003年的6.3倍，市级以上企业技术中心达到215家，研发中心达到412家。大力实施名牌战略，全市拥有中国名牌产品12个、中国驰名商标11个。

三、加快建设商贸都市，服务业发展实现新突破

积极发展物流、会展、金融、旅游等现代服务业，不断壮大批零贸易、住宿餐饮、交通运输等传统服务业。启动了圃田、航空港两个物流园区和11个现代物流中心建设，郑州铁路集装箱中心站、郑州国家干线公路物流港、郑州进口保税区等物流基础设施建设进展顺利。航空物流业发展迅速，先后开通了郑州至中东、北非的国际货运航班，鲲鹏航空公司总部入驻我市，春秋航空公司在我市设立分支机构。会展业发展迅速，硬件设施达到国内一流水平，成功举办了第二届中博会、中国国内旅游交易会及世界旅游城市市长论坛、第二届全球服务外包大会等大型展会。银行、证券、保险、期货、信托等金融产业快速发展，区域性金融中心建设迈出新步伐。汇丰银行、民生银行在我市设立分支机构；郑州商品交易所期货交易额达到15.6万亿元，是2003年的19.6倍。大力实施“万村千乡市场工程”、社区商业“双进”工程，不断完善以市级商业中心、区级商业中心和社区商业网点为依托的商贸流通网络。加快专业商品交易市场建设，12个批发市场交易园区的辐射力进一步增强。沃尔玛、家乐福、麦德龙、易初莲花、华润万家及大商、华联、苏宁等商业企业入驻我市。“家电下乡工程”全面推进。

四、加快发展现代农业，“三农”工作取得新成效

以增加农民收入为核心，以新农村建设为重点，着力推进农业和农村经济发展。五年来，市财政用于“三农”的总支出达到154.8亿元，年均增长35%。调整优化农业结构，大力发展特色农业，制定并实施了《郑州市现代农业发展总体规划》，积极构建都市型、近郊型、远郊型三个农业圈层，形成了一批特色农产品生产基地，畜牧业产值占农业总产值的比重达到44%。农业产业化经营水平不断提升，市级以上农业产业化龙头企业达到260家，农民专业合作社总数达到512个，建立各类农产品生产基地160万亩。农业综合开发取得新成效，改造中低产田30万亩，全市无公害、绿色、有机农产品基地面积达到206万亩。实施“百村示范、千村整治”工程，扎实推进新农村建设。建成新农村示范村103个，村容村貌显著改善。新建改建农村公路5486公里，新建户用沼气池15.5万座、大中型沼气池109座、集雨水窖4.5万个，解决了80万人的饮水安全问题，5.8万人实现脱贫。

五、不断提升城市综合承载能力，着力强化枢纽地位

以“四城联创”为载体，不断提升城市规划、建设和管理水平。认真贯彻落实《城乡规划法》，编制了《郑州市城市总体规划（2008-2020）》、《郑东新区总体规划》、《郑州市中心城区保护和改造规划》等一批发展规划，修订完善了《郑州市土地利用总体规划（2006-2020）》。进一步强化全国综合交通枢纽地位，高速铁路客运专线和铁路运输接点站、黄河公铁两用大桥、郑州高速铁路客运站、铁路集装箱中心站、火车站西出站口等一批重大项目开工建设，积极做好郑西客运专线、石武客运专线、京珠高速郑州至漯河段改扩建、连霍高速郑州至洛阳段改扩建的协调推进工作。加强城市基础设施建设，五年投入资金509亿元，打通断头路33条，拓宽改造道路12条，建成立交桥7座，实施雨污水管网和积水点改造工程215项，排水管网覆盖率达到85%，城市天然气普及率达到92%。优先发展城市公交，运营车辆达到4218台。加快城中村和旧城、危旧房改造步伐，共批准95个村（组）实施改造，拆迁面积达到1292万平方米。郑东新区“五年成规模”目标全面实现。建成区面积达到50平方公里，累计开工项目458个，完成固定资产投资617.7亿元，在建和建成房屋面积突破2000万平方米，入住人口突破30万人。郑州国际会展中心、河南省艺术中心和中央商务区内环30个高层项目全部建成并投入使用。龙湖南区、商住物流区、龙子湖区建设进展顺利。深入开展“城市管理年”活动，对道路交通、夜景照明、园林绿化、河湖水系、公共服务设施等进行改造提升。完成了十条精品街的综合整治。数字化城市管理系统投入运行。郑州植物园建成开放。深入开展爱国卫生运动，城市卫生环境明显改善。加快推进郑汴一体化，郑开大道建成通车，郑州和开封在公交、电信、旅游、商务等方面加快了融城步伐，郑汴产业带规划加快实施。航空港区建设稳步推进。中小城市和小城镇建设不断加强。

六、开展资源节约和环境保护，建设生态文明

深入开展流域治理、总量控制、生态建设及饮用水安全四大环保工程建设，加快形成节约能源资源和保护生态环境的产业结构、增长方式和消费模式。深入开展环境综合整治，加大对小火电、小煤矿、小水泥、小造纸和砖瓦窑厂的整治力度，关闭、淘汰污染严重和不符合产业政策的企业3000多家，拆除一大批燃煤锅炉和工业窑炉，各类燃煤机组全部完成了脱硫设施建设并稳定运行。集中整治违法用地、违法建设专项行动取得明显成效。完成了王新庄、五龙口、马头岗污水处理厂建设改造任务，六县（市）污水处理厂全部建成并投入运行，全市生活污水日处理能力达到98.5万吨。垃圾综合处理场、垃圾焚烧发电厂和医疗废物处置中心建成运营，日处理垃圾4710吨，生活垃圾无害化处理率达到80%，医疗废物集中处置率达到98%。大力实施森林生态城建设，新增造林84万亩。出台了《郑州黄河湿地自然保护区管理办法》，加大黄河湿地保护力度。积极实施生态水系建设，对金水河、东风渠、熊儿河等河流进行综合整治，市区河流初步实现了“水通、水清、水美”的目标。

七、发挥文化资源优势，推进文化强市建设

以项目建设和文化活动为载体，全面推进文化事业和文化产业发展。加快重点文化项目建设，中华炎黄二帝巨塑、黄帝故里改造等项目完工，商城遗址公园建设和嵩山申遗等项目进展顺利。实施文艺精品工程，《风中少林》、《禅宗少林·音乐大典》、《快乐星球》、《小樱桃》和《独脚乐园》等品牌名扬海内外。连续三年高规格举办黄帝故里拜祖大典，成功举办两届世界传统武术节和第十届亚洲艺术节，圆满完成奥运火炬传递。嵩山少林景区被评为国家首批5A级景区。2008年接待国内外游客3540万人次，实现旅游总收入342亿元，分别是2003年的3.5倍和3.1倍。完成了郑州歌舞剧院体制机制改革，实施了杂技团股份制改造。各县（市）区都建有文化馆、图书馆，建成社区文化中心100个和达到省级标准的农村文化大院500个。广泛开展科教、文体、法律、卫生进社区和文化、科技、卫生“三下乡”活动。

八、稳步推进各项改革，不断扩大对外开放

体制机制创新取得重大突破，开放型经济发展进入新阶段。国有企业改革取得实质性进展。中原制药厂、郑州轻汽、郑缆集团、白鸽集团和嵩岳集团所属部分企业的改制重组进展顺利，紫荆山百货大楼等国有商业企业成功引进了战略投资者，中小企业的改制工作基本完成，国有企业资产总额和经济效益稳步增长。完成了粮食流通体制改革。深化财税、金融、投融资体制改革。成立了郑州投资控股有限公司、郑州城建集团有限公司、郑州交通建设投资有限公司等8家投融资公司，进一步增强了政府的融资和调控能力。大力发展风险投资和创业投资，组建了郑州百瑞创新资本创业投资有限公司。加快企业上市步伐，五年内共有11家企业上市，全市上市公司达到20家。以乡镇机构、农村义务教育和县乡财政管理体制改革为主要内容的农村综合改革取得阶段性成果。非公有制经济发展迅速，占全市经济总量的比重达到60%，非公有制经济组织达到28.4万个，从业人员达到155万人。坚持“大招商、招大商”，不断创新招商方式，提高引资水平。2008年，全市实际使用境外资金14亿美元，是2003年的9.3倍，利用境内域外资金437亿元。对外贸易水平稳步提高，全市出口额达到29.5亿美元。鼓励企业开展对外投资，境外投资涉及30个国家和地区。

九、完善社会保障体系，人民生活水平进一步提高

切实保障和改善民生，积极构建民生福利指标体系，每年确定的与人民群众生活密切相关的“十件实事”均得到较好落实。五年来，市财政用于改善民生的总支出达到576.7亿元，年均增长30.1%。切实加强就业工作，累计实现城镇就业62.5万人、农村劳动力转移就业66.2万人、农村劳动力引导性培训69.5万人、专业技能培训34.3万人。养老、医疗、失业、工伤和生育等保险制度不断完善，参保总人数比2003年翻了一番。农村新型合作医疗制度全面推行，参合人数达到396.4万人，参合率达到96.4%。全面实施城乡低保制度，低保标准逐年提高。加强敬老院建设，农村五保对象集中供养率达到52%。探索出了流浪乞讨人员救助工作“郑州模式”。加强中小学校基础设施建设，市区新建、改扩建中小学校62所，为全市农村义务教育阶段中小学更新配备课桌椅22万套，妥善解决在郑务工人员的子女入学问题。大力发展职业教育，市职业教育基地建设进展顺利。高度重视市属高校建设，不断加大资金投入，不断提高办学层次和水平。加大保障性住房建设力度，累计开工建设经济适用房497万平方米，竣工338万平方米，“双困家庭” 廉租住房覆盖率达到100%。郑州人民医院公共卫生应急后备楼和病房楼、市中心医院病房楼等一批重点医疗卫生设施投入使用，完成了乡镇卫生院和村卫生室改造建设任务。实施食品药品放心工程和农产品市场准入制度，有效保证了人民群众饮食用药安全。积极构建人口和计划生育工作长效机制，稳定低生育水平，人口自然增长率控制在5‰以下。

十、加强民主法制和精神文明建设，社会大局保持稳定

自觉接受人大及其常委会的监督，认真执行人大及其常委会的决定、决议；加强与人民政协的协商，接受人民政协的民主监督。认真办理人大代表议案、建议和政协委员提案，不断提高办理质量。积极支持工会、共青团、妇联等人民团体开展工作，充分发挥其桥梁纽带作用。重视对台、侨务、民族和宗教工作。规范行政执法行为，提高依法行政水平。大力加强服务型政府建设，不断健全公共服务体系。按照《公务员法》有关规定，对一些重要岗位实行了干部交流轮岗。加强行政执行力和公信力建设，着力解决政令不畅、效能低下等问题。建立了政府新闻发言人制度，政务更加公开透明。加强行政审批制度改革，行政审批、许可事项从2308项减少到430项，设立了公开办事大厅和行政审批中心。扎实做好安全生产和信访稳定工作，社会治安防控体系不断完善，信访工作机制不断创新，应急管理水平不断提高，全市社会大局保持稳定。廉政建设和反腐败斗争取得新成效，政风行风明显好转。开展了深入学习实践科学发展观、解放思想大讨论和“两转两提”等主题活动，政府效能和公务员素质明显提高。大力弘扬“博大、开放、创新、和谐”的城市精神。社会主义荣辱观教育和精神文明创建活动深入开展，涌现出任长霞、朱和平、李隆等一批先进人物。双拥共建活动扎实开展。统计、审计、物价、人防、地方志、新闻出版、社会科学、档案管理、气象和地震监测等工作都取得了新成绩。

特别值得提出的是，在抗击冰雪和地震灾害中，全市人民大力发扬“一方有难、八方支援”和无私奉献精神，调集大批人力、物力和财力支援灾区，充分展示了郑州人民的深情厚爱。

各位代表！

过去的五年是我市综合实力显著增强的五年，是发展质量效益明显提高的五年，是城乡面貌发生巨大变化的五年，是人民群众得到实惠更多的五年，郑州在海内外的知名度和美誉度显著提高，人民群众的幸福感和自豪感显著增强。

回顾过去五年，我们深深体会到，经济社会能够实现跨越式发展，得益于我们始终坚持和把握了以下几点：

一是始终坚持把解放思想作为行动先导。按照“加快发展、维护稳定、为民谋利”的总体要求，解放思想，理清思路，强力实施跨越式发展战略。着力强化枢纽地位、优化工业结构、建设商贸都市、发展现代农业，保护改造中心城区、加快建设郑东新区、积极推进中小城市发展，努力建设文化郑州、生态郑州、平安郑州、和谐郑州。坚持大区域谋划、大产业支撑、大项目带动、大市场运作，加速生产要素向优势产业和重点区域聚集，加快资源优势向经济优势转变。新思想、新观念为推动我市经济社会跨越式发展增添了强大精神动力。

二是始终坚持把发展作为第一要务。发展是时代的主题，是破解一切难题的关键。无论外部环境如何变化，我们坚持发展不动摇。以科学发展观为指导，创新发展思路，破解发展难题，大力推进新型工业化和城镇化进程，统筹发展现代服务业和现代农业，大力开展资源节约和环境保护，努力实现经济社会与生态环境的协调发展。

三是始终坚持把自主创新作为关键环节。大力实施科技自主创新工程，提高自主创新能力，实现资源资本推动向科技推动转变、粗放管理向科学管理转变、经营产品向经营品牌转变。大力推进体制创新和管理创新，促进产学研紧密结合，集中力量攻克了一批带动性强、关联度大、作用突出的关键技术，在新型工业化道路上迈出了坚实步伐。

四是始终坚持把改革开放作为强大动力。改革力度不断加大，改革领域从商品市场向要素市场拓展，资本、人才、技术市场蓬勃发展；从竞争性领域向公用公共事业领域拓展，科技、文化、卫生、市政等领域的体制改革迈出新步伐；从经济领域向行政管理领域拓展，政府职能配置日趋合理，行政执行力显著增强。坚持实施开放带动主战略，把“引进来”和“走出去”紧密结合起来，利用两种资源、两个市场的能力不断增强，对外交流与合作空间不断扩大，对外开放的质量和水平显著提升。

五是始终坚持把执政为民作为根本要求。实现好、发展好、维护好广大人民群众的根本利益是政府工作的出发点和落脚点。我们把民生投入作为财政支出的重点，切实解决民生问题。加强公益事业和社会建设，大力发展科技、教育、文化、卫生等社会事业，让群众共享发展成果。努力维护社会公平正义，稳步提高城镇低收入者和广大农民的收入，积极构建完善的社会保障体系。

各位代表！

过去五年取得的这些成绩，是省委、省政府和市委正确领导的结果，是市人大、市政协大力支持的结果，是全市人民共同奋斗的结果。在此，我代表市人民政府，向工作在全市各条战线的广大人民群众和各界人士致以崇高的敬意！向关心、支持我市现代化建设的香港特别行政区同胞、澳门特别行政区同胞、台湾同胞、海外侨胞和国际友人表示衷心的感谢！

各位代表！

我们的工作还存在不少困难和问题，主要表现在：经济增长方式比较粗放，产业结构层次较低，第三产业发展相对滞后，节能减排形势严峻，土地、资源、环境等约束加剧；农业基础还比较脆弱，农民持续增收难度加大；群众对就业、就学、就医、收入分配、住房保障等方面还不尽满意；政府职能和作风转变还不到位，个别部门和工作人员依法行政观念和为民服务意识淡薄，政府效能还需要进一步提高。对此，我们将高度重视并认真加以解决，不辜负人民群众的期望。

今后五年经济社会发展的基本思路和主要任务

今后五年，是我市加快现代化、国际化、信息化和生态型、创新型城市建设，推进经济社会跨越式发展的重要时期，是全面规划建设大郑东新区的关键时期，我市将全面实现“十一五”目标，大力实施“十二五”规划。面对更加崇高的历史使命，面对更加复杂的经济发展环境，面对更加激烈的区域竞争态势，我们必须切实增强忧患意识、危机意识，进一步解放思想，更新观念，树立世界眼光，加强战略思维，以先进城市为标杆，自觉把郑州置于全省、全国乃至全球的发展大局之中去思考和谋划，努力走在全国省会城市的前列，当好中原崛起的排头兵。

我市经济社会发展的基本思路是：坚持以邓小平理论和“三个代表”重要思想为指导，深入贯彻落实科学发展观，积极推进改革开放，着力优化经济结构和提高经济增长质量，加快构建现代产业体系、现代城镇体系和自主创新体系，切实加强节能减排和生态环境保护，更加重视改善民生和促进社会和谐，加快经济大市向经济强市跨越，加快文化资源大市向文化强市跨越，提高工业化、城市化水平，提高社会事业发展水平，加快国家区域性中心城市建设，加快现代化、国际化、信息化和生态型、创新型城市建设。

我市经济社会发展的主要目标是：在优化结构、提高效益、降低消耗、保护环境的基础上，保持经济较快增长，到2013年经济总量超过5000亿元，人均生产总值超过60000元。地方财政一般预算收入达到430亿元，城镇居民人均可支配收入和农民人均纯收入分别达到23000元和12000元，人口自然增长率控制在6‰以内。

一是加快“三化两型”城市建设，提高核心竞争力。围绕提升城市经济、人民生活、社会发展和基础设施现代化水平，加快郑州现代化城市建设进程。主动参与全球合作与竞争，力争在优势领域达到世界先进城市水平。加快信息技术、信息产业和信息基础设施建设，促进信息技术在经济社会各个领域的广泛应用，不断推进城市数字化、网络化、智能化进程。加快形成节约资源和保护生态环境的产业结构、发展方式和消费模式，实现经济发展与生态环境相协调、人与自然相协调。完善创新体系，培育创新主体，增强创新活力，大力推动经济发展从要素驱动向创新驱动、从郑州制造向郑州创造转变。

二是建立现代产业体系，抢占竞争制高点。把加快经济结构调整作为提升经济竞争力、增创发展新优势的重要途径。进一步加快经济结构调整和发展方式转变，巩固农业基础地位，加快发展高新技术产业、先进制造业和现代服务业，加大交通、能源、水利、环保等基础产业建设力度。促进产业结构高级化、产业发展集聚化、产业水平国际化，加快形成具有较强辐射带动力和国际竞争力的现代产业体系，提高在更大范围内配置资源的能力和水平，在中原崛起乃至中部崛起中走在前列，在经济全球化和区域经济一体化的大格局中实现跨越式发展。

三是加快现代化基础设施建设，提升城市综合承载力。按照省委、省政府要求，高起点规划、高标准建设大郑东新区。高水平建设一批现代化、信息化相融合的战略性、枢纽型基础设施项目，重点加快郑州国际航空枢纽建设，加快构建以轨道交通和高速道路交通体系为骨干的现代大都市综合交通系统，打造服务全国、连通世界的综合交通枢纽，为提升城市综合承载力提供坚实支撑。

四是统筹城乡协调发展，加快城乡一体化进程。做好城乡发展规划，努力形成人口布局合理、产业体系完备、设施配套完善、生活环境优美、功能分区科学的复合型城乡空间布局，构建现代城镇体系。把加快形成城乡经济社会发展一体化新格局作为根本要求，着力推进城乡土地利用和规划一体化，着力推进城乡产业发展和产业布局一体化，着力推进城乡就业和社会保障体系一体化，着力推进城乡基础设施建设和公共服务一体化，着力推进城乡社会管理和社会事业发展一体化。

五是坚持改革创新，扩大对外开放。坚持不懈地把改革创新精神贯彻到经济社会发展的各个环节，积极推动重要领域和关键环节的改革攻坚。深化行政管理体制改革，推进经济领域的改革，创新社会管理机制，加快形成有利于推动跨越发展、促进社会和谐、符合国际规范的体制机制。大力支持非公有制经济发展，落实鼓励扶持政策，努力为非公有制经济创造更加宽松的发展环境。毫不动摇地坚持开放带动主战略，拓展对外开放的广度和深度，优化利用外资和对外贸易结构，推动企业开展跨国、跨地区经营，在更广领域和更高层次实现与国际市场的对接，切实增强国际经济合作能力。

六是加强社会建设，不断改善民生。坚持把改善人民生活作为发展的根本目的和持久动力。在经济发展较快、财政收入不断增加的情况下，拿出更多的财力物力，大力发展科技、教育、文化、卫生、体育等社会事业，促进人的全面发展，使广大人民群众学有所教、劳有所得、病有所医、老有所养、住有所居，努力建设创业之都、宜居城市、和谐家园，真正实现发展成果由人民共享。

2009年是我市实施跨越式发展新“三年行动计划”的第一年，是全面规划建设大郑东新区的开局之年，同时，也是我市经济发展面临较多困难和挑战的一年。我们将积极应对国际金融危机，坚定信心，振奋精神，沉着应对，共克时艰，坚决打赢这场“扩内需、保增长，调结构、上水平，抓改革、增活力，重民生、促和谐”的硬仗，努力实现全市经济社会平稳较快发展。

全市经济社会发展的主要目标是：生产总值增长12%左右；地方财政一般预算收入增长12%左右；全社会固定资产投资增长23%；社会消费品零售总额增长15%；市属及以下企业进出口增长13%；利用外商直接投资增长15%；居民消费价格涨幅控制在4%左右；城镇居民人均可支配收入和农民人均纯收入分别增长9%和10%；人口自然增长率控制在6‰以内；城镇新增就业12万人，城镇登记失业率控制在4.5%以内；单位生产总值能耗降低5.08%，化学需氧量和二氧化硫排放量分别下降4.5%和5%。

一、全面促进经济平稳较快发展

采取更加有力的措施，统筹兼顾、突出重点，着力保持经济平稳较快发展。

（一）扩大投资需求。强力推进跨越式发展新“三年行动计划”，在社会事业、农林水利、城市建设等领域谋划一批重大项目，优先落实配套资金，优先配置土地资源，优先办理各项手续，确保重大项目早开工、早见效。巩固和提升枢纽地位，配合做好郑西、石武铁路客运专线和郑州黄河公铁两用桥等项目建设工作，配合做好郑州铁路东站综合交通枢纽、郑汴城市轻轨等项目的前期工作，争取开工建设郑徐铁路客运专线、郑焦城际铁路、郑州至机场轻轨等项目。加快连霍高速郑州至洛阳段、京珠高速郑州至漯河段等扩建项目建设，争取郑州西绕城、铁魏公路建成通车。加快航空港核心区的规划建设，重点做好郑州机场第二航站楼、第二条跑道等配套项目建设。开工建设博爱至郑州天然气管道项目。推进综合能源基地建设，加快郑煤集团240万吨李粮店煤矿、150万吨樊寨煤矿建设，争取300万吨赵家寨煤矿建成投产；加快国电荥阳2×60万千瓦机组、华润电力登封二期1×60万千瓦机组项目建设，争取新密电厂二期2×100万千瓦机组早日开工，争取大唐巩义1×100万千瓦机组尽早具备核准条件。

（二）扩大消费需求。做好事业单位工资制度改革，继续落实企业最低工资制度，提高低收入者收入水平，增强居民消费能力。加快发展住宿餐饮、社区商业、物业、家政等服务性消费，大力开发旅游、文化、网络、动漫、体育健身等热点消费。继续实施“万村千乡市场工程”，加快配送中心建设，启动和撬活农村消费市场，农村连锁超市覆盖率达到80%以上。扩大“家电下乡”的范围和品种，加大财政对农民购买家电、通讯类产品的补贴力度。鼓励居民购买汽车、住房等大宗消费。清理消费领域相关规费，降低收费标准，优化消费环境。

（三）积极落实财政金融政策。优化财政支出结构，加大“三农”、社会事业、节能减排、环境治理等重点领域的支出，确保工资发放、改善民生、“十大实事”的资金需要。完善项目推介机制，加强银企合作，引导金融机构加大信贷投放力度，有效满足企业对信贷资金的正常需求。运用政府增信等手段，争取政策性银行扩大贷款规模。落实税收优惠扶持政策。创新融资方式，强化保险、票据、债券的融资功能。支持符合条件的企业发行债券。

（四）大力发展非公有制经济。贯彻落实支持非公有制经济发展的各项方针政策，制定更具操作性的政策措施，加强信用担保、社会化服务等体系建设，充分发挥行业协会在非公有制经济发展中的桥梁纽带作用。加大对非公有制企业尤其是技术含量高、有发展潜力企业的扶持力度，着力改善对非公有制企业的金融服务，切实解决融资难问题。

二、加快结构调整和发展方式转变

大力推进经济结构战略性调整，努力做到速度与质量、结构、效益相统一。

（一）推进工业结构优化升级。坚持走新型工业化道路，推进信息化与工业化相融合，振兴传统产业，壮大主导产业，培育新兴产业。研究制定汽车、铝工业、食品工业、纺织服装、信息等产业的振兴发展规划，加快建设百万辆汽车和现代煤化工产业基地，培育若干个年销售收入超千亿元的优势产业。大力发展硅材料与光伏、生物及新医药、数字化装备、新型功能材料及制品、光电技术等高技术产业。支持骨干企业以资产、资源、品牌、市场为纽带，实施跨地区、跨行业的联合、兼并、重组，提高企业规模经济水平和产业集中度，形成一批年销售收入超百亿元的龙头企业。加快推进高新区和新密光伏产业聚集区、威科姆国际生态软件园、河南省国家大学科技园、阿格斯能源薄膜太阳能电池、晶诚科技产能提升等高技术项目建设，加快推进海马（郑州）年产15万辆轿车、宇通集团5万台客车和3万台悬架、郑州日产第二工厂、少林汽车5000台大客车等汽车项目建设，加快推进郑煤机2000架液压支架、宇通重工500台环卫专用设备、大方桥梁160台大型施工装备等装备制造项目建设，加快推进思念50万吨速冻食品、三全食品基地、中粮集团国家农副产品精深加工基地、益海嘉里粮油深加工、双汇肉类和面制品加工及物流配送中心、河南中烟年产150万箱卷烟等食品项目建设。

（二）推进服务业产业升级。设立服务业发展引导资金，加快发展物流、会展、金融、科技中介、文化、旅游等现代服务业。进一步完善支持城区经济发展的配套政策，加快发展总部经济、楼宇经济、商圈经济。修编郑州商贸城建设规划，整合现有批发市场，改造升级农产品集贸市场，建设一批特色商业街区，构建现代化、国际化、专业化的市场体系。高水平编制郑州国际物流中心规划，加快郑州国家干线公路物流港、郑州航空港保税物流中心、郑州铁路集装箱中心站、九龙国际产业（物流）园区、中南邮政物流集散中心等重大物流项目建设，推进重点综合物流园区、重要冷链物流基地、粮食物流体系、出版物流配送体系建设，加快引进基地航空公司和大型国际航空物流企业。依托郑州国际会展中心，围绕汽车及零部件、物流、纺织服装等产业发展，筹办一批高层次展会和论坛。认真实施《郑州区域性金融中心建设规划纲要》，以郑东新区中央商务区为核心，吸引国内外金融、期货、保险机构在我市设立分支机构。支持郑州商品交易所扩大交易品种，推动期货市场稳步发展。抓好嵩山少林、黄帝故里、沿黄旅游带等旅游精品建设，努力打造具有世界影响力的旅游胜地。

（三）加快构建自主创新体系。出台提高科技自主创新能力、推动高新技术产业发展的政策措施，加大全社会研发投入。加快科技基础共用平台和重大科技专项建设，实施“创新人才培育计划”、“创新企业培育计划”和“创新型科技园区创建计划”，支持郑州高新区成为国家创新型科技园区。支持工程研发中心、核心试验室建设，构建区域创新体系。大力发展知识产权经济，扶持具有自主知识产权的技术、标准和品牌，加快形成产学研相结合的科技成果转化机制，促进自主知识产权成果产业化。

（四）大力发展产业集聚区。把产业集聚区作为优化经济结构、转变发展方式、实现集约节约发展的基础和关键。制定完善产业集聚区发展规划，把产业集聚区发展与城镇化相结合，促进城镇化与工业化协调发展。加快产业集聚区基础设施和结构调整项目建设，提升对产业、人口、环境的承载功能，推动企业向园区集中、园区向城镇集中、劳动力向城镇转移，逐步形成集聚促进就业、就业创造消费、消费拉动经济增长的良性发展机制。

（五）强化节能减排和生态建设。坚持生态优先，发展循环经济，推广绿色能源，倡导绿色交通出行方式，为发展腾出环境容量和承载空间。以创建国家环保模范城市为载体，落实节能减排目标责任制和“一票否决”制，确保节能减排任务圆满完成。加快城市污水处理厂建设，上半年完成城市污水处理厂化学需氧量和氨氮自动监控设备更新改造。加快推进国家生态园林城市建设，市区新增绿地500万平方米，开工建设西流湖生态园区。继续实施森林生态建设工程，完成造林16万亩，建成林业生态村100个，创建市级生态文明村30个。积极筹备2010年第二届中国绿化博览会。加强黄河湿地保护与恢复，启动郑州黄河国家湿地公园建设。加快实施生态水系规划，继续对七里河、魏河、十七里河、十八里河、索须河等河道进行治理，加快贾鲁河生态治理与人工湿地建设。在全省率先全部拆除集中供热区域内的燃煤供热小锅炉，对机动车尾气污染进行集中整治，确保城区空气质量优良天数达到310天以上。

三、促进农业和农村经济全面发展

高度重视并认真解决好“三农”问题，进一步巩固和加强农业基础地位。

（一）进一步加大“三农”投入力度。完善农业保护和补贴制度，大幅度增加对农村基础设施和社会事业的投入，大幅度提高政府土地出让收益、耕地占用税新增收入用于农业的比例，大幅度增加对农村公益性建设项目的投入，鼓励和引导社会资金投向农业农村。认真落实中央各项强农惠农政策，较大幅度提高粮食最低收购价，较大幅度提高良种补贴、农机具购置补贴、农资综合直补、农村沼气建设补助标准，落实好稻谷、大豆、油菜籽、棉花等农产品临时收储政策，切实调动广大农民的积极性。探索建立以财政为依托的地方性农业保险体系。大力实施土地开发整理，完成10万亩中低产田改造，以中牟为试点开展黄河滩地开发。

（二）加快农业结构调整。实施新一轮优势农产品区域布局规划，加快发展高效农业和休闲观光农业，在都市和近郊大力发展蔬菜、水果、花卉等主导产业。继续推进现代农业示范园区建设，现代农业示范区总数达到22个。加快推进现代畜牧业发展，优化现代畜禽生产结构，组织实施《郑州沿黄现代畜牧业发展规划》，抓好重大动物疫病防治和畜产品质量安全工作。设立农业发展专项资金，加大对农业产业化企业的支持力度，提升农业产业化经营水平。

（三）扎实推进新农村建设。继续实施新农村建设整村推进项目，完成示范村建设任务。加快发展农村教育、文化、卫生事业，加强农村道路、电网、通讯等基础设施建设。新建改建乡村公路500公里以上，新建集雨水窖5000个，解决农村30万人的饮水安全问题，新增农村沼气用户3万户。完成尖岗、常庄、唐岗、楚楼等大中型水库的除险加固任务。加大扶贫力度，完成10个贫困村的整村推进扶贫开发和1800户移民扶贫搬迁任务，实现1万人脱贫。完成南水北调工程库区移民安置工作。

（四）深化农村改革。按照依法自愿有偿原则，鼓励农民以转包、出租、互换、转让、股份合作等形式流转土地承包经营权，发展多种形式的适度规模经营。探索设立推进城乡一体化工作试点县（市），强力推进城乡一体化进程。充分发挥农信社、农发行、农行等涉农金融机构的作用，扩大村镇银行和小额贷款公司试点，完善支持农民小额贷款办法，探索建立乡镇小额贷款担保机构。加快集体林权改革。继续推进县乡财政管理体制改革，增强乡镇政府社会管理和公共服务职能。加强农村基层政权建设，完善村务公开和民主管理制度。深化殡葬改革，完成146个公益性公墓和290个骨灰堂的建设任务。

四、积极发挥中心城市带动作用

实施中心城市带动战略，加快城镇化进程，积极推进中原城市群一体化，加快建设国家区域性中心城市。

（一）规划建设大郑东新区。按照省委、省政府提出的郑汴新区规划布局，加快以郑东新区、经济技术开发区、郑汴产业带、航空港区、九龙国际产业（物流）园区和中牟县为主体的大郑东新区建设，把大郑东新区建设作为全市经济社会发展的“一号工程”，集全市之力加快推进。按照现代化、复合型、多功能、组团式、生态宜居的规划理念，采用国际邀标方式，上半年完成大郑东新区总体发展规划的编制工作。加快大郑东新区基础设施建设，推进以国际航运、铁路高速客运和集装箱转运为重点的现代化交通枢纽建设，开工建设郑少高速机场联络线、郑民高速郑州段、国道107线郑州段迁建工程，开工建设郑东新区至航空港区的快速通道，将紫荆山路向南延伸至航空港区，延伸中央大道至开封金明大道，延伸航海东路至省道223线。同时，加快水、电、气、暖及污水、垃圾处理设施建设。大力发展现代服务业、先进制造业、高新技术产业和都市农业，重点发展高端制造业、创新型产业和现代物流业。推进管理体制和机制创新，努力在财税体制、投融资体制、土地管理制度、公共服务体系等重点改革领域实现新突破。通过不懈努力，将大郑东新区建设成为现代复合型城区、综合改革核心实验区、对外开放示范区、环境优美宜居区，成为中原崛起的增长极、河南乃至全国内陆地区加快发展和改革创新的示范区。

（二）加强城乡规划与管理。贯彻落实《城乡规划法》，继续做好重点规划的实施和编制工作。认真组织实施《郑州市城市总体规划（2008-2020）》和《郑州市城市综合交通近期建设规划》等规划，加快推进《郑州市土地利用总体规划（2006-2020）》的修编工作，编制好《国家区域性中心城市建设规划》、《郑州市分区域控制性详规和专项规划》等规划。坚持建管并重、强化管理的原则，全面运行数字化城市管理系统，实施城市科学化、精细化管理。进一步完善城镇体系规划，构建中心城市、卫星城、重点镇、一般建制镇和农村居民点协调发展的五级城镇体系。

（三）加快城市基础设施建设。继续保护改造中心城区，建设一批精品街区，开工建设8座人行过街设施和8条下穿式隧道，对100条背街小巷实施改造，打通一批断头路，着力缓解交通拥堵问题。加快推进沙口路高架快速通道、京广路拓宽改造、中心区铁路跨线桥等工程，加快火车站西出站口建设，开工建设城市轨道交通一号线一期工程，完成二号线一期工程的前期工作，加快城市快速公交系统建设，对三环快速路实施升级改造，构建城市综合交通网络。加快推进雕塑公园、四环带状公园、大型苗圃建设和出入市口园林绿化整治，完善城市绿地系统。升级改造城市水、电、气、暖和信息网络，进一步增加集中供热面积，提高燃气化率。配合做好南水北调中线工程郑州段建设。

五、大力推动文化强市建设

推动文化发展与繁荣，提升文化软实力，为实现郑州经济社会跨越式发展提供文化支撑。

（一）积极发展文化事业。加快商城遗址公园、图书馆新馆、档案馆新馆、广电中心建设，开工建设郑州奥体中心、郑州市民健身中心等文化体育设施，办好第十届全市运动会。加大对文化事业的投入力度，加强重点文物和非物质文化遗产保护，全力推进嵩山历史建筑群申报世界文化遗产，支持登封申报国家级文化产业示范园区。加快推进文化惠民工程，新建50个乡镇（街道）综合文化站、800个行政村文化大院。继续实施农村电影放映工程。在700个行政村每村建设一个硬化的标准篮球场和两张室外乒乓球台。实现5万户无线数字电视信号“户户通”。

（二）加快发展文化产业。大力发展新闻出版、广播电视、创意、动漫、演艺等文化产业。重点抓好《禅宗少林·音乐大典》二期建设，扩大品牌影响。研究制定文化创意产业发展规划，加快建设中国郑州信息创意产业园，培育壮大创意产业。加快国家动漫产业发展基地（河南基地）和项目建设，支持小樱桃、天乐等动漫品牌做大做强。

（三）切实加强精神文明建设。加强社会主义核心价值体系建设，大力弘扬以爱国主义为核心的民族精神和以改革创新为核心的时代精神。积极开展建国60周年纪念活动。继续发扬“博大、开放、创新、和谐”的城市精神，加强社会公德、职业道德、家庭美德和个人品德建设。做好大学生和未成年人思想政治工作。加强图书、网络等文化市场管理。组织开展志愿服务活动，推动群众性精神文明创建活动蓬勃开展。

六、继续推进改革开放

总结改革开放30年经验，进一步解放思想、坚定信心，深化改革、扩大开放，增强经济社会发展的动力和活力。

（一）深入推进国有企业改革和战略重组。继续深化国有企业改革，建立和完善现代企业制度。鼓励优势企业兼并重组落后企业、困难企业，充分利用有效资产，稳定生产和就业。鼓励优势企业强强联合，提高规模效益，实现优势互补。鼓励关联产业、上下游企业的联合重组和战略合作，提高抗风险能力。

（二）深化投融资体制改革。全面推进政府投资项目代建制，提高政府投资效益。做好政府投资公司的运营工作，完成郑州商行重组、百瑞信托增资扩股和中小企业担保公司战略投资者引进工作，加快组建市农村商业银行和农村合作银行。强化对重点上市后备企业的培育和督导，推动更多的企业上市融资。创新融资方式，大力发展风险投资和创业投资。

（三）积极实施开放带动战略。围绕培育壮大战略支撑产业、骨干优势企业、重大产业基地和产业集聚区，推出一批重大引资项目，重点做好国内外大企业的引进工作，鼓励外资企业参与企业重组和传统产业技术改造。鼓励与沿海发达地区建立产业转移联盟，承接产业集群式和链式转移。认真落实各项出口退税政策和扶持政策，切实缓解出口企业面临的突出困难。推进加工贸易转型升级，扩大高技术和机电类产品出口。设立服务外包发展专项资金，大力发展服务外包产业。积极争取郑州成为国家汽车及零部件出口基地。支持企业开展境外投资、工程承包和劳务输出。

七、加强以改善民生为重点的社会建设

以办好省、市确定的“十大实事”为重点，切实解决好人民群众最关心、最直接、最现实的利益问题。

（一）进一步扩大就业。实施更加积极的就业政策，新增城镇就业12万人、农村富余劳动力转移就业10万人，完成农村劳动力技能培训6万人以上。大力发展劳动密集型产业，加快中小企业发展，努力创造更多的就业岗位。坚持发展就业和全民创业相结合，制定鼓励全民创业的政策措施，完善创业服务体系。做好高校毕业生和复转军人就业安置工作。安排农民工创业扶持专项资金，制定并落实农民工返乡创业的政策措施，促进农民工就业。加大对就业困难人员、零就业家庭的帮扶力度。

（二）大力发展教育事业。围绕巩固义务教育、突出职业教育、提升高等教育水平的原则，加快教育事业发展。市区新建、改扩建中小学15所，逐步缓解大班额问题。逐步理顺中小学校管理体制。加大城乡教师交流和对口支援工作力度，努力实现教育均衡发展。积极化解农村“普九”债务，提高农村中小学校生均公用经费标准。着力改善农村办学条件，落实农村义务教育经费，完成农村初中实验室建设任务。大力发展学前教育。为特殊教育学校配备现代化教学设备。加快建设国家职业教育改革实验区，推进郑汴产业带职教基地建设，对全市农村考上中等职业学校的学生每人每年补助学费1000元。大力发展高等教育，推进郑州师专申报郑州文理学院。

（三）不断完善社会保障体系。进一步健全城乡居民基本养老保险制度，扩大参保覆盖面，全年全市参保人数达到35万人。提高企业退休人员养老金标准，人均月增加120元。完善和落实城乡居民最低生活保障制度，加强对城乡特困户、低收入家庭等困难群体的社会救助，加大对高等院校家庭经济困难学生的生活补助力度。把在我市稳定就业半年以上失去工作的农村务工人员纳入失业登记管理，发放一次性生活补助。进一步提高城乡低保、农村五保、医疗救助等补助标准，扩大农村低保范围，做到应保尽保。为全市低保家庭的精神病患者每人每年提供1200元补助，为全市无劳动能力、无生活来源、无法定抚养人或赡养人的残疾人每人每月提供300元补助。巩固完善新型农村合作医疗制度，报销比例统一提高10%，封顶线由3万元提高到5万元。加强社区建设，进一步提高社区服务、保障和管理水平。加快社会福利事业发展，大力推进社会化养老服务。新建改扩建25所敬老院，农村五保户集中供养率提高到55%。加快社会保障性住房建设，开工建设经济适用房150万平方米、廉租房15万平方米、周转房20万平方米。

（四）提高卫生服务水平。建立涵盖妇幼保健、疾病预防、社区卫生服务等医疗卫生服务的信息网络体系。大力推行“片医负责制”，中心城区覆盖人口达到140万人以上。构建社区卫生服务圈，让社区群众15分钟内能够到达服务中心。推进农村卫生服务体系建设，新建1000个标准化村卫生所，为1318个村卫生所配备医疗设备，为全市96个乡镇卫生院各配备一辆急救车。加强农村卫生人才队伍建设，全面推进城乡医院结对帮扶工作。加快市妇女儿童医院建设，加快市精神病医院改扩建、市疾控中心和市七院整体迁建等项目。增加重大传染病、慢性病和职业病防治专项投入，为全市1万名白内障患者免费实施复明手术。

（五）认真做好人口和计划生育工作。稳定低生育水平，综合治理出生人口性别比偏高问题，提高出生人口质量。新建扩建35个乡镇计生服务站，开展免费婚前检查。继续落实计划生育家庭特别救助制度，独生子女父母奖励标准统一提高到40元，对农村只有一个子女或两个女孩且年满60周岁的夫妇，奖励扶助标准由每人每年600元提高到1000元。

（六）扎实做好安全生产和信访稳定工作。加大行政问责力度，严厉打击违法违规行为，强化煤矿瓦斯和水害治理，加强非煤矿山、化工、烟花爆竹、道路交通、消防等重点行业的安全治理，坚决遏制重特大事故发生。加大食品药品安全监管力度，城市和农村食品市场抽检合格率分别比上年提高2个和3个百分点。加强群众和信访工作基层基础建设，着力解决突出问题，完善矛盾纠纷调处机制，有效解决群众合理合法的利益诉求。积极开展“平安建设年”活动，完善社会治安防控体系，加强社会治安综合治理，严厉打击各类犯罪行为，为人民群众营造安全、祥和的社会环境。

继续做好国防动员、人民防空工作，加强全民国防教育和民兵预备役建设，深入开展双拥共建活动。巩固平等、团结、互助、和谐的社会主义民族关系，发挥宗教界人士和信教群众在促进经济社会发展中的积极作用，充分发挥工会、共青团、妇联等人民团体的桥梁纽带作用。扎实做好统计、审计、外事、侨务、对台事务、社会科学、地方志、档案管理、气象和地震监测等工作。继续做好对江油四镇的对口援建工作。

努力建设服务型政府

在复杂多变的经济形势下，全面实现保增长、保民生、保稳定的目标任务，政府肩负着重要责任。我们将以深入学习实践科学发展观为动力，扎实推进“两转两提”，切实提高社会管理和公共服务水平，努力建设人民满意的服务型政府。

一、加快转变政府职能

进一步加强制度建设，健全行政决策、执行、咨询、反馈、监督等方面的程序和制度。积极稳妥推进政府机构改革，上半年完成市级政府机构改革，年底前完成县级政府机构改革。切实加强政府精细化管理，创新政府管理模式。深入开展“企业服务年”活动，主动为企业做好服务。大力推进行政审批制度改革，再取消和调整一批审批项目。健全岗位责任制、服务承诺制、限时办结制、首问负责制、效能考评制和失职追究制。

二、严格依法行政

自觉接受人大及其常委会的法律监督、政协的民主监督以及司法监督。认真贯彻落实《国务院关于加强市县政府依法行政的决定》和《郑州市关于加强市县政府依法行政的若干意见》。坚持科学立法、民主立法，改进政府立法方式。规范社会秩序，规范行政执法行为，确保严格执法、公正执法、文明执法。加强全社会法制建设，完善依法行政监督机制，做好依法行政的指导协调工作。认真开展行政复议、行政执法监督检查和投诉处理工作。

三、大力推行政务公开和科学决策

认真贯彻落实《政府信息公开条例》，保障群众的知情权、参与权、表达权和监督权。进一步拓展政务公开的领域和内容，完善和增加政务公开载体，继续推进电子政务建设。坚持决策程序民主化，建立健全重大问题集体决策制度、决策责任追究制度和决策合法性论证制度。大力推行听证制度，建立健全重大决策专家咨询制度。

四、切实转变工作作风

牢记全心全意为人民服务的宗旨，深入开展调查研究，做到问政于民、问需于民、问计于民，使政府工作思路、工作部署、政策措施更加符合实际。加强督促检查，确保各项重大决策落实到位。树立正确的政绩观，建立健全符合科学发展观要求的政府工作考核体系，加强目标管理，推行行政问责和政府绩效管理制度，不断提高行政执行力。进一步提高工作效率，提升服务质量，提高行政效能，优化发展环境。牢记“两个务必”，厉行节约、勤俭办事，严格控制一般性支出，对公务车辆经费、会议经费、公务接待费、出国出境经费等支出实行“零”增长，建设节约型机关。加强反腐倡廉建设，坚决惩治和有效预防腐败。

各位代表！

站在新起点，实现新跨越，责任重大，使命光荣。让我们更加紧密地团结在以胡锦涛同志为总书记的党中央周围，在省委、省政府和市委的正确领导下，解放思想，开拓创新，齐心协力，扎实工作，为把郑州建设成为现代化、国际化、信息化和生态型、创新型城市而努力奋斗！
